# Screening for osteoporosis: A systematic assessment of the quality and content of clinical practice guidelines, using the AGREE II instrument and the IOM Standards for Trustworthy Guidelines

**DOI:** 10.1371/journal.pone.0208251

**Published:** 2018-12-06

**Authors:** Lamia M. Hayawi, Ian D. Graham, Peter Tugwell, Said Yousef Abdelrazeq

**Affiliations:** 1 Pallium Canada, Ottawa, ON, Canada; 2 Bruyère Research Institute, University of Ottawa, Ottawa, ON, Canada; 3 School of Epidemiology and Public Health, University of Ottawa, Ottawa, ON, Canada; 4 Clinical Epidemiology Program, Ottawa Hospital Research Institute, Ottawa, ON, Canada; 5 School of Nursing, Queen’s University, Kingston, ON, Canada; 6 Centre for Global Health, University of Ottawa, Ottawa, ON, Canada; Van Andel Institute, UNITED STATES

## Abstract

**Background:**

Numerous clinical practice guidelines (CPGs) are published to guide management of osteoporosis. Little is known about their quality or how recommendations have changed over time.

**Objective:**

To systematically assess the quality and content of the guidelines on screening for osteoporosis, using the Appraisal of Guidelines for Research and Evaluation (AGREE II) tool, and the Institute of Medicine (IOM) standards for trustworthy guidelines.

**Methods:**

We conducted a systematic search for osteoporosis CPGs published between 2002–2016, using multiple databases and guideline websites. Two reviewers appraised the quality of eligible CPGs using the AGREE II. High quality CPGs were considered if they scored ≥ 60 in four or more domains including the domain for rigor of development. Non-parametric tests were used to test for the change of quality over time. One reviewer assessed the guidelines with IOM standards. We summarized the different evidence grading systems and extracted and compared the recommendations.

**Results:**

A total of 33 CPGs were identified. The mean scores for AGREE II differed by domain (range: 42% to 71%). CPGs scored higher on domains for clarity of presentation, scope and purpose, and rigor of development. CPGs scored lower on domains for stakeholder involvement, editorial independence and applicability. Assessment of CPGs by IOM standards showed that CPGs scored better on standards for systematic review, establishing evidence foundation and rating strength of recommendation, articulation of recommendation, and establishing transparency. While scored lower on standards for updating, external review, and the development group composition. There was no difference in AGREE II and IOM defined guidelines’ quality before and after the introduction of the two tools (P values >0.05). The IOM identified four more guidelines as high quality compared to the AGREE II. Examining these additional guidelines indicated that the two tools may give conflicting results especially for the rigor of development domain. Recommendations in certain areas showed substantial differences between guidelines.

**Conclusion:**

Osteoporosis screening CPGs are of variable quality, and their recommendations often differ. Guideline quality as measured by AGREE II and IOM standards has not improved overtime. Guideline developers should work together to improve the quality and consistency of recommendations to improve the likelihood that their guidelines will be used in practice.

## Background

Osteoporosis is a disease characterised by low bone mass and deterioration of the bone tissue structure leading to increased bone fragility and liability to fractures [1]. These fractures usually result from low mechanical forces such as a fall from standing height or less, that usually don’t cause a fracture [2]. The most common sites of these fractures are in the spine, hip, and wrist [2]. Worldwide, osteoporosis leads to nearly 9 million fractures annually [1].

Low bone mass is a major risk factor for fractures, however, there are many other factors that contribute to osteoporosis including age, sex, previous fractures, family history of osteoporosis, use of systemic glucocorticoids, excessive alcohol and smoking [3]. The prevalence of osteoporosis rises rapidly with age; thus the incidence of fractures is predicted to increase with the increased longevity of the population [4].

Patients who suffer osteoporotic fractures are at higher risk of morbidity and mortality; in the Canadian Multicentre Osteoporosis Study (CaMOS), both men and women had increased incidence of death of 23.5% (20/85) after hip fracture [5]. Additionally, these fractures cause acute pain and loss of function, and hip fractures nearly always lead to hospitalisation. Recovery is slow and rehabilitation is usually insufficient, leading to decreased quality of life and burden on caregivers [6]. The economic cost of these fractures is high; Hopkins et al 2016, estimated that it costs the Canadian health system more than $ 4 billion per year [7].

Osteoporosis is usually diagnosed based on bone mineral density (BMD) measurement by dual energy x-ray absorptiometry (DXA). A T-score of -2.5 standard deviation below the expected mean value for a young white female adult, is considered diagnostic [8]. Since low bone density is not the only risk factor for osteoporosis, a variety of tools were developed to aid in diagnosis and decision to decide the initiation of pharmacological treatment. These tools incorporate the main risk factors for osteoporosis with or without the DXA testing T-score; such tools are CAROC, FRAX, QFracture, and others [9].

The aim of screening is to diagnose those at risk for fractures to prevent them from occurring, in addition to prevent the risk of re-fracture in patients who sustained a previous fragility fracture. Many pharmacological and non-pharmacological treatments are available and have proven efficacy [10].

Clinical practice guidelines (CPGs); which are defined by the Institute of Medicine as “*Statements that include recommendations intended to optimize patient care that are informed by a systematic review of evidence and an assessment of the benefits and harms of alternative care options*” [11]. Guidelines should consist of recommendations for assessment and management of specific diseases based on the latest evidence. Clinicians make countless number of decisions each day and they do not have the time to consider all the underlying evidence for these decisions; CPGs can do this for them [12]. Hence, CPGs are intended to transfer evidence into practice, decrease variability in clinical practice and decrease costly and avoidable harms or mistakes [13].

The number of guidelines have increased substantially; at the time of this study the CMA (Canadian Medical association) infobase included approximately 1,200 CPGs [14]. However, the effect of CPGs on improving the process and outcome of care have varied widely [15]. Furthermore, even for well- developed guidelines, their adoption and use is not an automatic process and depends greatly on the dissemination process and how they are implemented [16,17].

In past years, there were many efforts to improve the development of guidelines, and to standardize the method of development. The AGREE II instrument was developed by an international team of researchers to define the essential components of a good guideline [18]. A review by Vlayen et al 2005, reported that AGREE II is the most validated compared to 24 other tools, with easy scored numerical scales [19]. Additionally, different frameworks to grade the level of evidence have been released; the Grading of Recommendations Assessment, Development and Evaluation (GRADE) system has and was adopted by many organizations and guideline developers such as NICE, and WHO [20]. Recently, the GRADE Working Group has developed a framework “the Evidence to Decision (EtD)”, to assist the process of progressing from evidence to making clinical recommendations, coverage decisions, and health system or public health recommendations and decisions [21].

Guidelines for screening of osteoporosis have been developed by many agencies and organizations. Previous literature reported that they conveyed mixed messages to primary care physicians [22]A systematic review by Cranney et al., 2002 of CPGs for postmenopausal osteoporosis released between 1998 to 2001 found that these guidelines were of low quality [23]. Those which were of acceptable quality were most commonly developed by researchers in the United States, one was from Ontario, Canada, and two were from the United Kingdom.

Most studies from different North American and European countries indicated that a high proportion of individuals whom are at risk of fragility fractures are not being screened, which reflects the low adherence of physicians to the CPG [4,6,24–26].Thus, determining the quality of current guidelines is important and to our knowledge there is no recent review of the quality of CPGs relevant to osteoporosis screening. Therefore, we conducted a systematic review to assess the quality of guidelines using the AGREE II and the IOM standards, determine whether osteoporosis guidelines quality has improved over time, summarize the grading systems for level of evidence and strength of recommendations, and to compare the recommendation for their consistency/concordance.

## Methods

This systematic review followed the Cochrane Methodology [27], to identify, and select the CPGs and the Preferred Reporting Items for Systematic Reviews and Meta-Analyses (PRISMA) to guide the reporting of this review [28]. Ethics approval was not required as this work was based on systematic literature review.

### Search strategy and data extraction

A systematic search for relevant guidelines was performed between January 2002 and September 2016 using the following databases: Embase, Medline, Pubmed, Canadian Medical Association Infobase, National Guidelines Clearinghouse, and Guidelines International Network (http://www.g-i-n.net/). Some well-known guidelines developers; Excellence (NICE), and the Scottish Intercollegiate Network (SIGN), were also searched, in addition to reviewing references of each guideline for other relevant guidelines.

Key words used for the MEDLINE search can be found in S1 Table which are modified according to the indexing systems. Inclusion criteria were; CPGs with recommendations for adult population; guidelines for screening of osteoporosis with or without treatment. The applied period was from 2002–2006, and the guidelines should be intended for health professionals. Language restrictions were not applied, however non-English texts were later excluded. Guidelines were also excluded, if they only addressed glucocorticoid induced osteoporosis or specific diseases or conditions such as hyperthyroidism, inflammatory bowel disease, celiac disease, and post-gastrectomy states. Additionally, we excluded position papers and consensus papers since they are not equivalent to guidelines.

Screening of titles and abstracts and then full texts were carried out by one reviewer (LA), a second reviewer (SY) screened a sample of 100 full text articles to check the accuracy of screening. Guidelines that did not meet the inclusion criteria were excluded. One reviewer (LA) extracted the following information: Guidelines titles, Authors, publication year, country, the organization that produced the guideline, and main key recommendations with the systems used for assigning level of evidence and strength of recommendations.

### Guidelines appraisal and data analysis

#### Quality assessment

The AGREE instrument is a tool; which was developed in 2003 and then was updated in 2010 to the AGREEII instrument [18]. The purpose of the tool is to provide a systematic framework to assess the methodological rigour of guideline quality and a methodological strategy for developing the guidelines [18]. The tool consists of 23 items which are grouped into 6 domains (scope and purpose, stakeholder involvement, rigour and development, clarity of presentation, applicability, and editorial independence) [S2 Table]. Items are rated on 7-point scale ranging from 1 (absence of them) to 7 (exceptional quality of item).

Two appraisers (LA and SY) appraised each guideline independently using the AGREE II. Only information included in the released guideline or their references were used for the appraisal process, i.e., the reviewers did not refer to additional supporting documents that were published separately, unless explicitly indicated by the CPGs.

Domains scores were calculated by summing up item scores within each domain for each reviewer, then standardising it as a percentage of maximum possible score

$$\mathrm{Scaled}\;\mathrm{domain}\;\mathrm{score}=\frac{obtained\;score-minimum\;possible\;score}{maximum\;possible\;score-minimum\;possible\;score}x\;100$$

Descriptive statistical analysis were conducted, and agreement between reviewers was assessed by using two-way, random, single unit, absolute agreement intra-class correlation coefficients ICC (2,1) [29]. In addition, we obtained Cohen’s weighted kappa to compare with ICC, using squared weights, since we have an ordinal scale (AGREE II 1–7), [29]. The degree of reviewer agreement was categorized based on Cicchetti (1994) ICC<0.40 poor; 0.40 to 0.59 moderate; 0.60–0.74 good; 0.75–1.00 excellent [30].

The Institute of Medicine (IOM) in the United States in 2011 developed standards “Clinical Practice Guidelines We can Trust”; to aid developers in producing quality evidence based guidelines[11]. The resulting instrument has eight standards (Establishing transparency, management of conflict of interest, guideline development group composition, systematic review intersection, establishing evidence foundation, articulation of recommendation, external review, and updating) with 20 sub-criteria or attributes, with no proposed numerical scoring [IOM with all subcriteria in S3 Table [31].

Differences from the AGREE II tool domains are summarized in S4 Table. IOM has separate standards for external review and for updating the guidelines, while, there are no standards that assess applicability, or resource implications.

We scored each of the 20 subcriteria by 1, or 0; and a standard was considered to be met, if more than half of the sub-criteria were fulfilled. For example; for standards with 3 sub-criteria, 2 or more need to be fulfilled to consider that standard is met. One reviewer (LA) used this tool to appraise the guidelines.

We hypothesized that the quality of guidelines has changed and improved over time especially after updating the AGEEII tool in 2010 and the introduction of IOM standards in 2011. So, we to tested our hypothesis by using a non-parametric test, the Wilcoxon Rank-Sum test (Mann-Whitney test) to test for statistical significant differences in domain scores between CPGs published before and in/after 2010 (AGREE II update) and total IOM scores before and after IOM development [32]. For IOM standards, a Chi square test was used to find if the proportion of guidelines meeting each IOM standard has improved after the IOM development. If expected cell counts were less than five, then Fisher’s exact test is used instead [32]. P values less than 0.05 were considered statistically significant.

#### Comparison of AGREEII tool and IOM

Because the two tools don’t have the same items, we compared the two tools by determining whether both tools identify the same guidelines as being of high quality. For that purpose; with the AGREE II, guidelines were considered of high quality, if they scored ≥ 60% in 4 or more domains including domain 3 for rigor of development, since we consider this domain as an important part of guideline quality. This approach in identifying high quality guidelines has been reported in other studies assessing the quality of CPGs [33–35]. Similarly, with the IOM standards, we defined high quality guidelines if 5 or more standards were met including standards 4 and 5 (*CPG systematic review intersection*, *and Establishing evidence foundations for and rating strength of recommendations respectively*). We didn’t compare statistically if the difference between the two tools in identifying the high quality guidelines is significant, since our sample size is low. Yet, we examined the different identified high quality guidelines to find out which domains or areas differ between the two tools, and which tool may give a better trusted results. We were only able to identify one study that compared guidelines quality using both instruments. Bennett et al 2016 [36], compared IOM instrument with the AGREE II, but they changed the method of AGREE II scoring. We opted not to do the same, in order not to change the scoring of the AGREEII, as this may decrease the result’s validity.

Analyses were preformed using Microsoft Excel and SAS 9.4 statistical package, except Inter-rater reliability (ICC & weighted kappa) was performed using R statistical software [37].

## Results

### Search results

**Fig 1:**
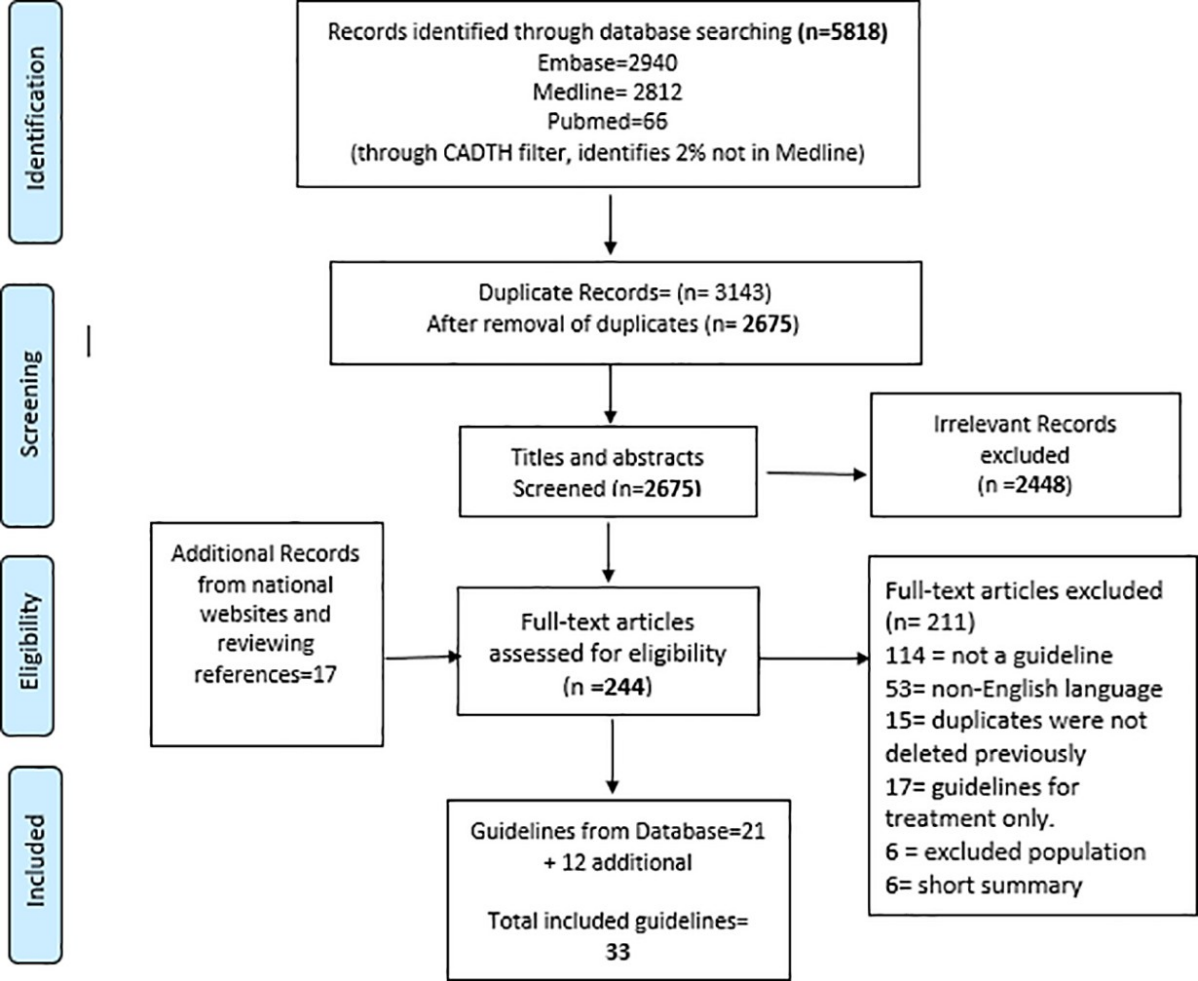
Prisma Flow chart for the selection of the guidelines.

A total of 5,818 records were identified from our electronic systematic search of databases, of which 3,143 were excluded as duplicates, and 2,448 were found to be irrelevant after screening the titles and abstracts. We found an additional 17 records from national guideline websites and hand searching references of identified guidelines. Thus, a total of 224 records were screened as full text, and 211 were excluded for a variety of reasons. Finally, 33 final guidelines were eligible for assessment (21 guidelines from databases, and 12 from other sources) presented in Table 1. The screening process for CPGs is presented in the Prisma Flow diagram [Fig 1].

**Table 1:** Details of the retrieved guidelines, 2002–2016.

	Title	Country	Organization	Year	Accessed through
**1**	2002 clinical practice guidelines for the diagnosis and management of osteoporosis in Canada [49]	Canada/ Ontario	Osteoporosis Canada	2002	Database
**2**	Screening for Osteoporosis in Postmenopausal Women: Recommendations and Rationale [50]	USA	US Preventive Service Task Force (USPSTF)	2002	Database
**3**	Prevention of osteoporosis and osteoporotic fractures in postmenopausal women: recommendation statement from the Canadian Task Force on Preventive Health Care [43]	Canada/ Ontario	Canadian Task Force on Preventive Health Care	2004	Database
**4**	Lebanese Guidelines for Osteoporosis Assessment and Treatment [51]	Lebanon	The American University of Beirut	2005	Database
**5**	Guidelines for diagnosing and prevention and treatment of osteoporosis in Asia [42].	South Korea, India, China, Malaysia, Singapore, Sri Lanka, Philippines	Not reported	2006	Database
**6**	Screening for Osteoporosis in Men: A Clinical Practice Guideline from the American College of Physicians [52].	USA/ national	American College of Physicians	2008	Database & National Guideline Clearinghouse
**7**	Clinician’s Guide to Prevention and Treatment of Osteoporosis. [47]	National	National Osteoporosis Foundation	2008	Reference
**8**	First Update of the Lebanese Guidelines for Osteoporosis Assessment and Treatment [53].	Lebanon	The American University of Beirut	2008	Database
**9**	Osteoporosis MOH Clinical Practice guidelines [54].	Singapore	Ministry of Health	2009	IOF
**10**	2010 Clinical Practice guidelines for the diagnosis and management of osteoporosis in Canada: a Summary [40].	Canada/ Ontario	Osteoporosis Canada	2010	Database
**11**	Medical Guidelines for Clinical Practice for the Diagnosis and Treatment of Postmenopausal Osteoporosis [55].	USA	American Association of Clinical Endocrinologist AACE	2010	National Guideline Clearinghouse
**12**	Clinical guideline for prevention and treatment of osteoporosis in postmenopausal women and older men [38].	Australia	The Royal Australian College of General Practitioners.	2010	IOF
**13**	NOFSA guideline for diagnosis and management of osteoporosis [56].	South Africa	The National Osteoporosis Foundation of South Africa (NOSFA)	2010	IOF
**14**	2011 Guidelines for the diagnosis and treatment of osteoporosis in Greece [57].	Greece	Greece National Medicine Agency	2011	Database Search
**15**	Screening for osteoporosis: the US Preventive Services Task Force Recommendations Statement [58].	USA/ National	USPSTF	2011	database and Nat. guideline clearinghouse
**16**	Guidelines for Clinical Care Ambulatory Osteoporosis Guideline Team Lead Ambulatory Clinical Guidelines Oversight Osteoporosis: Prevention and Treatment [59].	USA/ Michigan	University of Michigan Health System	2011	National Guideline Clearinghouse
**17**	Taiwan osteoporosis practice guidelines [60].	Taiwan/ National	Bureau of Health Promotion, Department of Health, ROC (Taiwan)	2011	National Guideline Clearinghouse
**18**	Osteoporosis: Diagnosis, Treatment and Fracture Prevention [44].	Canada/BC	British Columbia Medical Association	2012	IOF, CPG infobase
**19**	Clinical guidance on the management of osteoporosis, 2012 [61].	Malaysia	The Malaysian Osteoporosis Society	2012	Reference
**20**	Osteoporosis: Assessing the risk for fragility fracture [9].	UK/England	NICE	2012	IOF and NICE website
**21**	Osteoporosis in Men: An Endocrine Society Clinical Practice Guideline [62].	USA	The Endocrine Society	2012	Database
**22**	Diagnosis and treatment of Osteoporosis [45]	USA/ Minnesota	Institute for Clinical System Improvement	2013	National Guideline Clearinghouse
**23**	Clinical Practice Guidelines on Postmenopausal osteoporosis: Executive Summary and Recommendations [63].	India	Indian Menopause Society	2013	Database
**24**	Clinician’s Guide to Prevention and Treatment of Osteoporosis [48].	National	National Foundation of Osteoporosis	2014	Database
**25**	Osteoporosis in Menopause[64]	Canada/	Society of Obstetricians and Gynaecologists of Canada	2014	Database
**26**	Guidelines for the diagnosis, prevention and management of osteoporosis [65]	UK/National	National Osteoporosis Guideline Group (NOGG)/UK	2014	Database
**27**	Management of osteoporosis and the prevention of fragility fractures A national clinical guideline [46]	Scotland/UK	Scottish Intercollegiate Guidelines Network (SIGN)	2015	IOF
**28**	A summary of the Malaysian Clinical Guidance on the management of postmenopausal and male osteoporosis, 2015 [66].	Malaysia	The Malaysian Osteoporosis Society	2015	IOF
**29**	2015 Guidelines for Osteoporosis in Saudi Arabia: Recommendations from the Saudi Osteoporosis Society [67].	Saudi Arabia	Saudi Osteoporosis Society	2015	Database
**30**	Osteoporosis clinical guideline for prevention and treatment: Executive Summary [68].	UK/National	National Osteoporosis Guideline Group(NOGG)/UK	2016	Google Scholar
**31**	Guidelines for the diagnosis, prevention and management of osteoporosis [69].	Italy	Italian Society for Osteoporosis, Mineral metabolism and Bone Diseases (SIOMMMS)	2016	Database
**32**	Diagnosis and management of Osteoporosis [70]	Canada/ Alberta	Toward Optimized Practice/Alberta	2016	Database, CPG infobase
**33**	Clinical Practice guidelines for the diagnosis and treatment of postmenopausal osteoporosis-2016 [71].	USA/National	American Association of Clinical Endocrinologists and American College of Endocrinology	2016	Database

### Quality of CPGs based on the AGREE II Score

The standardized domain scores, the weighted kappa, and the Intra-class correlation coefficient (ICC) values for each guideline are depicted in Table 2. The agreement between the two reviewers is ranging between (ICC = 0.50–0.93) except for the Australian guidelines 2010 [38], which indicates according to Cicchetti’s cut off points a moderate to excellent agreement [30]. We also presented the weighted kappa agreement scores which agrees with the ICC, however, ICC is a preferred method for inter-rater reliability for ordinal scales [39].

**Fig 2:**
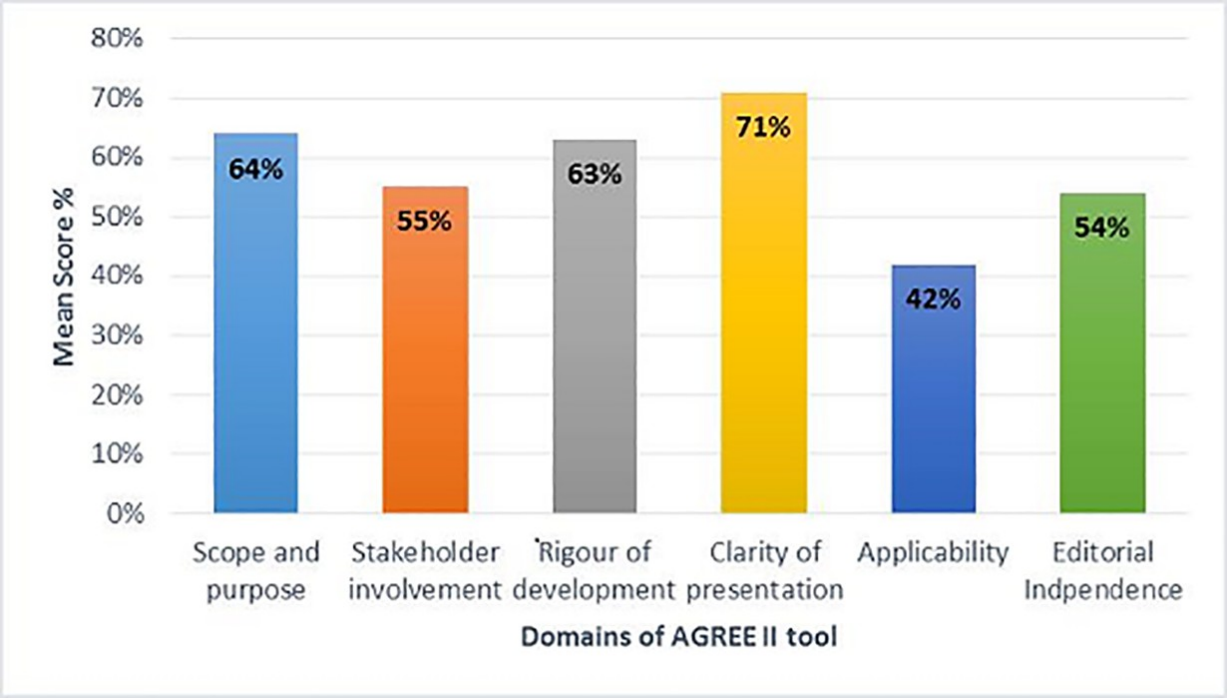
AGREE II mean standardised score for each domain for CPGs (2002–2016).

The descriptive statistics for each domain is presented in Table 3 with the mean standardized scores for each domain is shown in Fig 2

**Table 2:** AGREE II standardised domain scores and inter-reliability tests for CPGs from 2002–2016.

Guideline	1.Scope and purpose	2.Stakeholder Involvement	3.Rigour of development	4.Clarity of presentation	5.Applicability	6.Editorial Independence	Intra-class Correlation (ICC) %	Weighted Kappa %
**Osteoporosis Canada 2002** [49].	83	69	81	92	52	75	50	50
**US Preventive Services Task force 2002** [50].	61	36	75	50	13	33	93	92
**Canadian Task Force on Preventive Health Care 2004** [43]	31	31	41	58	17	46	62	61
**American University of Beirut Medical Center 2005** [51].	72	44	64	61	23	0	88	87
**Guidelines in Asia 2006** [42].	17	22	18	42	33	0	72	70
**the American College of Physicians guidelines 2008** [52].	83	58	55	69	33	79	67	66
**National Osteoporosis Foundation 2008** [47].	58	61	33	69	27	42	89	88
**First Update of the Lebanese Guidelines 2008** [53].	78	47	70	64	27	8	85	84
**Singapore Clinical Guidelines 2009** [54]	47	92	64	78	56	13	89	65
**Osteoporosis Canada 2010** [40].	100	75	83	75	48	50	76	76
**American Association of Clinical Endocrinologist AACE 2010** [55]	78	56	65	72	33	46	71	70
**The Australian Guidelines 2010** [38]	78	92	98	97	88	96	44	45
**South Africa guidelines 2010** [56]	75	89	90	97	46	46	74	73
**Greece National Medicine Agency 2011** [57]	36	22	20	44	17	50	75	74
**USPSTF 2011** [58]	78	42	83	67	50	83	60	60
**University of Michigan Health System guideline 2011** [59].	67	44	70	69	40	58	86	84
**Taiwan Osteoporosis guideline 2011** [60]	67	75	92	94	54	96	61	60
**British Columbia Medical Association 2012** [44]	36	8	33	58	52	13	78	78
**The Malaysian Osteoporosis Society Guideline 2012** [61].	100	81	88	92	88	88	67	67
**NICE guidelines 2012** [9]	81	100	94	92	88	96	70	70
**The Endocrine Society 2012** [62]	42	33	71	67	40	92	73	72
**Institute for Clinical System Improvement guideline, 2013** [45].	**92**	**100**	**91**	**97**	**81**	**100**	**74**	**74**
**Indian Menopause Society, 2013** [63]	72	50	84	78	58	33	68	67
**National Foundation of Osteoporosis** **2014** [48]	86	42	25	69	27	54	82	82
**the Society of Obstetricians and Gynaecologists of Canada** [64]	50	42	60	78	8	25	91	90
**National Osteoporosis Guideline Group (NOGG)/UK 2014** [65]	36	17	10	33	23	71	75	74
**The Malaysian Osteoporosis Society guideline, 2015** [66]	58	28	57	61	23	79	78	78
**Scottish Intercollegiate Guidelines Network (SIGN) guideline, 2015** [46]	81	100	99	86	83	83	86	86
**2015 Guidelines for Osteoporosis in Saudi Arabia** [67].	61	50	58	78	21	42	83	82
**National Osteoporosis Guideline Group(NOGG)/UK, 2016** [68].	50	67	61	75	58	100	79	78
**Italian Society for Osteoporosis, Mineral metabolism and Bone Diseases 2016** [69]	36	28	50	50	15	0	75	74
**Alberta Guidelines 2016** [70]	58	42	22	69	27	13	91	91
**American Association of Clinical Endocrinologist AACE 2016** [71]	75	58	74	75	44	58	75	74

-ICC interpretation: ICC<0.20 poor; 0.21–0.40 fair; 0.41–0.60 moderate; 0.61–0.80 good; 0.81–1.00 very good. -CPGs which are highlighted with grey are considered high quality guidelines (achieved 60% or more in domain rigour of development with 3 other domains).

**Table 3:** Descriptive statistics summarizing the AGREE II domain scores of all guidelines (2002–2016).

GREE II Domains	Mean	SD	Median	Min, Max
**Scope and purpose (%)**	64.33	20.95	67	17–100
**Stakeholder involvement (%)**	54.57	25.96	50	08–100
**Rigor of development (%)**	63.00	25.53	65	10–99
**Clarity of presentation (%)**	71.30	16.52	69	33–97
**Applicability (%)**	41.75	22.83	40	08–88
**Editorial dependence (%)**	53.57	32.57	50	00–100

**Domain 1: Scope and purpose.** This domain assesses the overall aim and objectives of the guidelines, the health questions and the target population. The mean score with (SD) for this domain was 64.33% ± (20.95%), most guidelines did well in this domain; however, their scores varied widely, ranging from (17% to 100%). Osteoporosis Canada 2010 [40], and the Malaysian guidelines 2012 [41]; both scored 100%. The worst scores were for the Asian guidelines 2006 [42] (17%), and for the Canadian Task force, 2004 [43](31%).

**Domain2: Stakeholder involvement.** This domain focuses on the participation of the professional experts, preferences of target population in the guideline development and whether target users are clearly defined. The mean score with (SD) was 54.57% ± (25.96%) with a very wide range (8% for British Columbian CPG [44] to 100% for NICE, Institute of Clinical System Improvement (ICSI) and SIGN [9,45,46]. Two thirds of guidelines (21/33) scored below 60% in this domain as most of the guidelines didn’t seek the views of the other stakeholders such as patients, public, payers, and policy makers in guideline development.

**Domain 3: Rigour of development.** This domain includes eight items that assess the systematic methods used for gathering and synthesizing of the evidence, and formulating the recommendations, the external peer review process and the procedure for updating the guideline. The mean score for this domain with (SD) was 63% ± (25.53%). Some important guidelines that are constantly cited in the field of osteoporosis such as the National Osteoporosis Foundation guidelines of 2008, and 2014 [47–48]scored low; 33%, and 22% respectively, as they did not report any systematic approach for developing their guideline. Most guidelines didn’t describe the process of updating clearly.

**Domain 4: Clarity of presentation.** This domain covers the language, structure and format of the guideline, and emphasizes on the clarity of the recommendations. The mean score with (SD) was 71.30% ± (16.52%), indicating that most guidelines had clear recommendations.

**Domain 5: Applicability.** This domain considers the barriers and facilitators to implementation of the guideline, approaches to increase uptake, resource implications of applying the guideline, and monitoring of the uptake or adherence to the guideline.

Consistently across all the CPGs, this was the lowest scored domain with means score and (SD) of 43.00% ± (24.45%), and a range between (8%-88%). Only 3 guidelines (SIGN [46], NICE[9] and ICSI[45]) reported monitoring and auditing criteria.

**Domain 6: Editorial independence.** This domain relates to the formation of recommendations under unbiased influence of the funding body, and with no competing interests of the developers. The mean score with (SD) was 53.57% ± (32.57%), almost half of the guidelines either didn’t provide statement about the funding or the competing interests. Some guidelines [42,51,69] scored 0% on this domain.

Table 4 presents the results of the testing for change in quality for each domain after the development of AGREE II (≤ 2010 vs ˃ 2010). All domains did not improve after the AGREE II update (p values >0.05).

**Table 4:** Change in AGREE II domain score (2002–2016), pre-AGREE II (n-13), post-AGREE II (n = 20).

AGREE II Domains	Mean (Median) pre- AGREE	Mean (Median) post- AGREE	Mean Difference	P value	95% Confidence Intervals
**Scope and purpose**	0.66 (75)	0.63 (64)	-0.03	0.58	-0.11–0.22
**Stakeholder involvement**	0.59 (58)	0.51(43)	-0.08	0.33	-0.11–0.28
**Rigor of development**	0.64 (65)	0.62 (65)	-0.02	0.97	-0.18–0.21
**Clarity of presentation**	0.71 (69)	0.71(72)	0	0.81	-0.14–0.11
**Applicability**	0.37 (33)	0.44 (42)	0.07	0.46	-0.25–0.10
**Editorial dependence**	0.41 (46)	0.61 (64)	0.20	0.06	-0.50–0.00

### Quality of CPGs determined by the IOM standards

**Fig 3:**
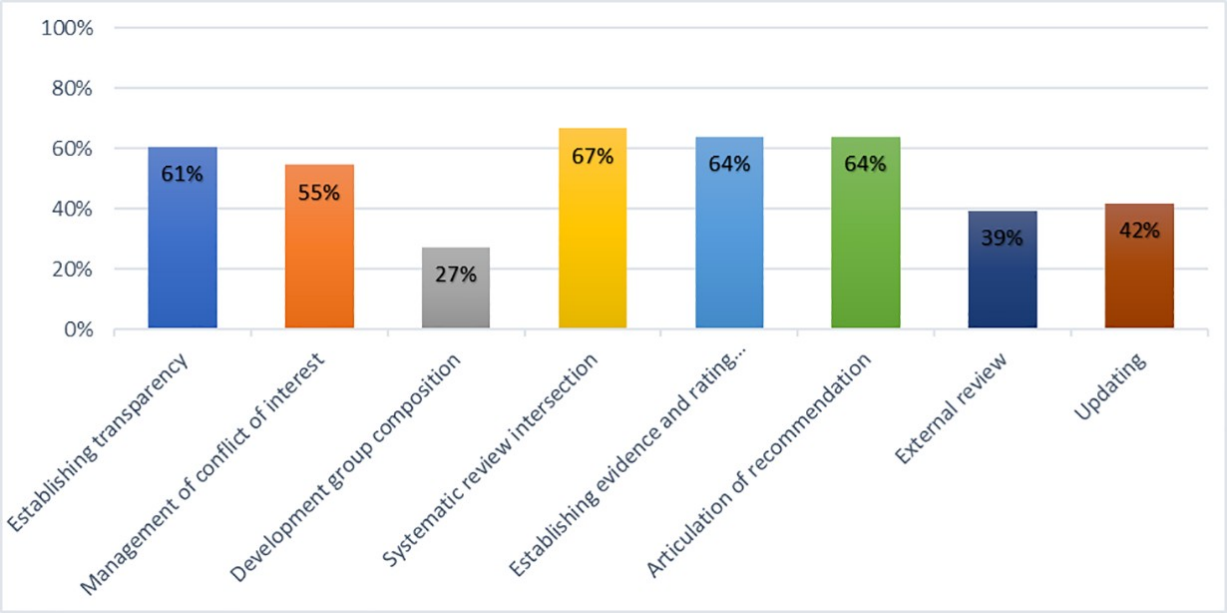
The percentage of CPGs meeting the IOM standards for Trustworthy Guidelines.

The eight IOM standards with their scores are presented in the S5 Table. The mean overall score is 4.18 out of 8 major standards. Four guidelines fulfilled all the eight standards (Australia CPGs 2010 [38], NICE 2012[9], ICSI CPG 2013 [45], and SIGN CPG 2015 [46]). Fig 3 shows that more than 60% (67%, 64%, 64%, 61%) of guidelines met standards for systematic review intersection, establishing evidence and rating strength of recommendation, articulation of recommendation, and establishing transparency respectively. 55% of CPGs met Standard 2 for Management of conflict of interest; which assesses the disclosure of conflict of interest of the guideline development group. Less than half of the guidelines fulfilled standards for external review and updating the guidelines (42%, 39%) respectively. The least fulfilled standard is for the development group composition, as only 9 (27%) of CPGs met this standard; most guidelines did not involve patients or public representatives or involve strategies to increase participation of patients or consumers.

Table 5 shows that there is no significant statistical difference in the proportion of guidelines that met the IOM standards after its introduction except for the systematic review standard in which the result of significance test was borderline (difference = -32%, P = 0.05). Furthermore, we assessed the difference in the total IOM score (out of 8) for the guidelines before and after the IOM publication and found statistically insignificant change (mean difference = -1.08, P = 0.74, 95% CI = -2 to 2). In essence, the quality of osteoporosis guidelines as assessed by the IOM standards instrument has not changed since the release of the tool in 2011.

**Table 5:** Proportion difference in CPGs meeting the IOM standards (2002–2016). Pre-IOM (n = 17), post-IOM (n-16).

IOM Standards	Count and (%) of CPGs pre-IOM	Count and (%) of CPGs post- IOM	Difference in %	P value For Chi square
**1: Establishing transparency**	11 (65%)	9 (56%)	-9%	0.63
**2: Management of conflict of interests**	7 (41%)	11 (69%)	28%	0.12
**3: Guideline development group composition**	4 (24%)	5 (31%)	7%	0.71
**4: systematic review section**	14 (82%)	8 (50%)	-32%	0.05
**5: Establishing evidence for and rating strength of recommendations**	13 (76%)	8 (50%)	- 26%	0.11
**6: Articulation of recommendations**	12 (71%)	9 (56%)	-15%	0.39
**7: External review**	5 (29%)	8 (50%)	3%	0.23
**8: Updating**	7(41%)	7 (44%)	3%	0.88

### High Quality CPGs Identified by both AGREE II Instrument and IOM Standards

When applying our criteria for determining a high quality guideline, AGREE II identified 13 such guidelines (39%), and the IOM identified 15 (45%). 11 CPG were identified by both tools[9,38,61,40,45,46,49,56,58–60]. The National Osteoporosis Guideline Group guideline (NOGG), 2016 [68] was additionally found by the AGREE II as high quality, but not according to IOM standards. The IOM standards identified other additional guidelines as high quality: The US Preventive Service Task Force (USPSTF) 2002, American College of Physicians 2008, Singapore 2009, and Malaysia 2015 [50,52,54,66] which were not identified as high quality by the AGREE II instrument.

We examined the four additional high quality guidelines, which were identified by IOM, and found that both tools may produce different conclusions in regard to the domains of systematic review, and strength of evidence, which most clinicians may consider most important in deciding which recommendation to follow. For instance, in two guidelines (American College of Physicians 2008 [52], and the Malaysian guidelines 2015 [66]), the domain for rigour of development in AGREE II scored <60%, while the matching IOM standards ‘4’ and ‘5’ were fulfilled. This is because questionnaires’ content that assess these domains differ between the two tools (Table 1); the external review and updating are included as part of the rigor of development in AGREE II, while they have a separate standard in IOM. Additionally, the items for systematic review section and the quality of evidence for formulating the recommendations are more detailed in the AGREE II compared to the IOM. Therefore, we found that the AGREE II would give lower scores if the systematic review process or methodology were not reported in detail in the guideline.

We tested whether the percentage of high quality guidelines differed before and after the introduction of AGREE II and the IOM standards (30.77% vs 40% after the AGREE and IOM introduction respectively). The percent change was 9.23% and statistically not significant. (% change = 9.23%, P = 0.72).

### Evidence and recommendations grading systems in use by guideline developers

The guidelines used different grading systems for determining the level of evidence and different systems for assigning the strength of recommendations. Table 6 summarises these systems. Nine out of the 33 guidelines didn’t assign grades for level of evidence or strength of recommendation to their recommendations. These were the Canadian Task Force 2004 [43], Lebanese CPG 2005 [51], Guidelines in Asia 2006 [42], NOF 2008 [47], Update of the Lebanese CPG 2008 [53], Greece 2011 [57], British Columbia CPG 2012 [44], NOF 2014 [48], Alberta CPG 2016 [70]. Seven guidelines used the Grading of Recommendations, Assessment, Development and Evaluation (GRADE) system [20], which many organizations are adopting or modifying for their use. These are: American College of Physicians 2008 [52], South Africa CPGs 2010 [56], NICE 2012 [9], The Endocrine Society 2012[62], Indian Menopause Society 2013 [63], Institute for Clinical System Improvement 2013 [45], and the Scottish Intercollegiate Guideline Network 2015 (for recommendations strength) [46]. There is a worldwide agreement to use this system and many respectful organizations moved their systems to GRADE such as SIGN recommendations [46], ICSI [45], and NICE [9].

**Table 6:** Grading systems used for determining the level of evidence and strength of recommendations.

**Guidelines**	**Grading System used for level of evidence**	**Description**	**System for strength of recommendations**	**Description**
**Osteoporosis Canada 2002** [49].	Same levels for diabetes guidelines by Meltzer et al 1998 [74] (it depends on the type of study i.e. interventional, prognostic…etc.)	1,2, 3, 4. For diagnosis and prognosis studies 1+, 1, 2+, 2, 3, 4, 5, 6 for intervention and treatment studies	same as diabetes guideline	A, B, C, D
**US Preventive Services Task force 2002** [50].	The USPSTF criteria	High, Moderate, Low	The USPSTF grades	A, B, C, D, I = (Insufficient)
**Canadian Task Force on Preventive Health Care 2004** [43]	Levels of evidence were not assigned		Which system they followed was not reported	
**American University of Beirut Medical Center 2005** [51].	Levels of evidence were not assigned but only reported The Royal College of Physicians, London Criteria,2000.		Royal College of Physicians, London 2000.	A, B, C
**Guidelines in Asia 2006** [42].	Levels of evidence were not assigned		Grades of strength were not assigned	
**the American College of Physicians guidelines 2008** [52].	American college of physicians grading system which is adapted from the GRADE system [52]	High, Moderate, Low	American college of physicians grading system which is adapted from the GRADE system [52]	Strong, or Weak
**National Osteoporosis Foundation 2008** [47].	Not assigned		Not assigned	
**First Update of the Lebanese Guidelines 2008** [53].	Were not assigned		Not assigned.	
**Singapore Clinical Guidelines 2009** [54]	Adapted from SIGN [46]	1++,1+,1-,2++,2+,2-, 3, 4	The previous SIGN grades before moving to GRADE	A, B, C, D
**Osteoporosis Canada 2010** [40].	Same criteria as 2002 guidelines[49], and diabetes guidelines in 1998	1,2, 3, 4. For diagnosis and prognosis studies 1+, 1, 2+, 2, 3, 4, 5, 6 for intervention and treatment studies	Same as evidence	A, B, C, D
**American Association of Clinical Endocrinologist AACE 2010** [55]	Adapted from AACE protocol by Mechanick et al [75]	1 1 2 2 2 2 3 3 3 3 4	Adapted from AACE protocol by Mechanick et al [76]	1 = strong 2 = intermediate 3 = weak 4 = no evidence
**Australian Guidelines 2010** [38]. [46]	NHRMC evidence matrix and grades of recommendation [38]	A, B, C, D	NHRMC	A, B, C, D
**National Osteoporosis Foundation of South Africa CPG (NOFSA), 2010** [56].	GRADE system [20]	High, Moderate, Low, very Low	GRADE [76]	1 = strong “we recommend” 2 = Weak “we suggest”
**Greece National Medical** **Agency Guideline, 2011** [57].	Not assigned		Not assigned	
**USPSTF 2011** [58].	USPTSF levels of certainty	High, Moderate, Low	USPSTF	A, B, C, D, I = Insufficient
**University of Michigan Health System Guideline 2011** [59].	Rating was assigned, but the source of the system was not reported	I, II, III	Grades assigned but the source was not reported	A, B, C, D
**Taiwan osteoporosis practice guidelines 2011** [60].	SIGN system of evidence	1++,1+,1-,2++,2+,2-, 3, 4	Grades were created by the developers [60]	A, B, C, D
**British Columbia Medical Association 2012** [44].	Levels were not assigned		Not assigned	
**The Malaysian Osteoporosis Society Guideline 2012** [61].	Adapted from the National Guideline Clearinghouse [77]	I, Ia, II, IIb, III. IV	Modified SIGN by Harbour et al 2001 [78]	A, B, C
**NICE guidelines 2012** [9]	They modified the GRADE system [80]	GRADE+ review of the quality of cost-effectiveness studies and don’t provide a summary labels for the quality of evidence across all outcomes.	Used a special NICE way of wording [79]	Most recommendations should start with an action verb ‘offer’, ‘consider’, ‘measure’, ‘advise’, ‘discuss’, ‘ask’, ‘about’, ‘commission’.
**The Endocrine Society 2012** [62]	GRADE [20]	High, Moderate, Low Very Low.	GRADE [76]	1 = Strong “we recommend”. 2 = Weak “we suggest”
**Institute for Clinical System Improvement guideline (ICSI), 2013** [45].	Transition between the ICSI system and GRADE	High, Moderate, Low	Same as evidence	Strong, or weak.
**Indian Menopause Society, 2013** [63]	GRADE [20]	High, Moderate, Low, Very Low	GRADE [76]	Strong = “recommend” Weak =“ suggest”
**National Foundation of Osteoporosis (NOF),** **2014** [48]	Levels were not assigned		Grades were not assigned	
**the Society of Obstetricians and Gynaecologists of Canada (SOGC), 2014** [64]	Canadian Task Force on preventive health Care 2003 [81]	I, II-1, II-2, II-3, III	Same as evidence	A, B, C, D, E (against), I (insufficient)
**National Osteoporosis Guideline Group (NOGG)/UK 2014** [65]	Not assigned		Not assigned	
**The Malaysian Osteoporosis Society guideline, 2015** [66]	National guidelines clearinghouse criteria, (Shekelle et al 1999 [77])	I, Ia, II, IIb, III, IV	Modified SIGN by Harbour et al 2001 [78]	A, B, C
**2015 Guidelines for Osteoporosis in Saudi Arabia** [67].	London College of Physicians, 2002[81]	Ia, Ib, IIa, IIb, III, IV	London College of Physicians, 2002[81]	A, B, C
**Scottish Intercollegiate Guidelines Network (SIGN) guideline, 2015** [46]	SIGN level of evidence [46]	1++,1+,1-,2++,2+,2-, 3, 4	GRADE System	-Strong -Conditional -Good Practice Points
**National Osteoporosis Guideline Group(NOGG)/UK, 2016** [68].	Levels assigned but the system was not reported	Ia, Ib, IIa, IIb, III, IV	ABC grades were assigned	A, B, C
**Italian Society for Osteoporosis, 2016** [69]	Levels of evidence were assigned but system was not reported	1, 2, 3,	Assigned	A, B, C, D
**Alberta Guidelines 2016** [70]	Not assigned		Not assigned	
**American Association of Clinical Endocrinologist (AACE), 2016** [71]	According to AACE protocol [75]	1 1 2 2 2 2 3 3 3 3 4	Grades were according to AACE protocol [75]	1 = strong 2 = intermediate, 3 = weak 4 = no evidence

### Summary of comparison between the recommendations

We reviewed the recommendations of 21 most recent and updated guidelines (2010 onward) in major areas of management of osteoporosis [“fracture risk estimation tool before BMD testing”, “BMD testing before risk estimation”, “when to start treatment?”, “considering 2 sites for BMD testing”, and “BMD testing after treatment”].

#### Use of fracture risk estimation tool before BMD testing

Many tools have been developed by researchers incorporating many factors to estimate fractures risk over 5 or 10 years [72];FRAX was developed by the WHO in 2008, and it is one of the most used and validated tool [72]. It estimates the 10-year absolute risk of fracture based on many risk factors, with or without Bone mineral density testing. The fracture risk probability varies by country, therefore, it was calibrated using country specific data where fracture rates and deaths are known [73].

There was a substantial variation between recommendations in whether to use a tool (FRAX) before BMD testing for risk assessment. This variation did not show any pattern of difference or similarity based on high and low quality guidelines, and mostly differed by region or country Four guidelines were not clear in their recommendations whether to use FRAX first or BMD testing (Society of Obstetrician and Gynecologist of Canada ‘SOGC’ 2014[64], the Saudi Arabia CPG[67], the Italian CPG 2016 [69], and the American Association of Endocrinologist 2016 [71]).

We found variations between guidelines in the same country; for instance, in Canada, variability between provinces is evident; British Columbia recommends using FRAX to determine the need for DXA [44]. While in Ontario, BMD testing is performed before FRAX, which is used afterwards to calculate the fracture risk estimation [40], and in Alberta, they use osteoporosis self assessment tool (OST), to decide the need for BMD testing. [70].

BMD testing before FRAX:Only four CPGs (Osteoporosis Canada 2010 [40], Australia 2010[38], National osteoporosis society of South Africa 2010[56], and Greece 2011[57]) recommend BMD testing before FRAX risk estimation especially for those <65 years of age. However, the recommendation for using FRAX is governed by the availability of country specific data and in countries like India where such data is not available, FRAX can’t recommended to be use neither before, nor after BMD testing.

#### When to start treatment

There was no pattern of consistency or similarity between high or low quality guidelines, or in relation to the date of publication. In general, we found four approaches for setting intervention thresholds. The first approach in countries with no country specific FRAX data, the treatment is based on the T-score value of BMD testing, such as India and South Africa CPGs [42,56]. As a second approach; some guidelines (all the Canadian CPGs) apply a fixed threshold of FRAX probability score that can be used for men and women irrespective of age. A 10-year risk of major osteoporotic fracture of ≥ 20% is the intervention threshold in most guidelines (Osteoporosis Canada 2010 [40], Taiwan 2011 [60], British Columbia 2012 [44], SOGC 2014 [64], and Alberta 2016 [70]). The third approach is using the T-score threshold, and if it is at the osteopenia level (T-score between -1 to -2.5), then FRAX score threshold is applied to decide treatment CPGs that used this approach are: Greece CPG2011 [57], Endocrine Society 2012 [62], Institute for clinical system improvement guideline 2013 [45], NOF 2014 [48], The Malaysian osteoporosis society 2015 [66], and the Italian Society 2016 [69].

The last fourth approach is applying of an intervention threshold which is dependent on age; the National Osteoporosis Guideline Group (NOGG) has set a threshold of intervention at each age level after 40 years, provided through a chart with or without BMD testing [65,68]

#### Sites for BMD testing

There was little discrepancy between guidelines’ recommendation in this area.

#### BMD testing after treatment

The period to assess BMD has varied between CPGs ranging between 1–2 years or 2–3 years or sometimes up to 8 years. However, most guidelines were in agreement in reporting that there is lack of evidence for the optimum period or the benefit of repeating BMD and that area is controversial.

## Discussion

We systematically identified and assessed 33 guidelines for screening for osteoporosis published between 2002–2016 from 13 countries, using the AGREE II instrument and the IOM standards for trustworthiness, which are developed to appraise the quality of CPGs. Our findings reveal that there has been marked variability in the compliance to the criteria of the AGREE II tool and the IOM standards by these guidelines.

An examination of the mean of AGREE II domain scores showed that the highest mean domain scores were for Clarity of Presentation and Scope and Purpose, while the lowest mean scores were for Applicability and Editorial Independence domains. This was consistent with other reviews in other topics [34,35,82,83]. The applicability domain reflects the implementation of the guideline however; most guidelines didn’t give advice on how the guideline should be implemented. One of the reasons for this might be that most guidelines don’t have experts in knowledge translation and economists within their development group which could advise on strategies for implementation and assessing economic barriers.

Regarding the domain of stakeholder involvement; most guideline developers didn’t seek the views and preferences of their target population especially patients in guidelines development and even when they did they were vague about the process. This is worth considering by all guideline developers, since a patient centred approach to health care with shared decision making is associated with better application of the guidelines and improved health care[84]. The domain of rigor of development which is considered of importance to guidelines quality scored only 60% of the guideline, thus, one third of CPGs had poor development methodology. This differs from the results of the previous review of postmenopausal osteoporosis CPGs by Cranney et al 2002 [23], in which the average score was almost 23%. Nonetheless, this review covered 2001–2002 published guidelines, so including more guidelines in our review may resulted in higher and more accurate mean score. Leslie & Schousboe 2011 reviewed 8 osteoporosis guidelines in an illustrative way rather than a systematic approach [85]. They assessed the quality of these guidelines using 7 items from the 23 AGREE II items and a different method for scoring, therefore, we could not compare our AGREE II results to theirs [85]. Nevertheless, we agree with their findings of conflicting recommendations in the same areas in these guidelines. Similarly, our review agrees with a review by Lewiecki M. in regards to the variability and conflicting recommendations especially in areas of evaluation and treatment of osteoporosis [86].

By assessing the compliance of guidelines to the criteria of the IOM standards, we found that 64%- 67% of guidelines fulfilled the standards for establishing evidence, strength of recommendations, and systematic review standards. However, most guidelines fell short in involving patients and public representatives in their guideline development and didn’t adequately describe the method for external review. Though, the IOM standards were developed in 2011, we found few studies that assessed the quality of CPGs using these standards[36,87,88][.These studies used different methodology, making it difficult to compare results. Reams et al 2013 [88], assessed the quality of guidelines for oncology using the IOM tool; their findings were similar to ours in particular to the lower score in guideline development group composition, yet, our study found a better performance on standards 3, 4 and 5 compared to their study. We found that the application of IOM standards to assess the guidelines quality is challenging, since no scoring system is assigned to the criteria and some of the sub-criteria are vague or partially fulfilled. This was also found in the study by Kung et al using the IOM standards, they excluded many items reporting that they were “vague and subjective”[87].

In our study, we found that the compliance of guidelines to the criteria of both tools (AGREE II and IOM) showed no change between 2002–2010 and 2011–2016, allocating the time of the release of both tools as the comparing point. This finding aligns with previous studies evaluating CPGs over time[82,87,89–91]while it conflicts with others [92–94];Armstrong et al. 2016 conducted a quality assessment and structured analysis for recommendations of physical activity and safe movement in osteoporosis guidelines [93,35]. They found an improvement in the quality guidelines over time. Nevertheless, they just reported the average AGREE II scores without a proper statistical test to find if this improvement were statistically significant. In our review, we also found more guidelines of high quality after 2010, yet, it was statistically insignificant improvement (p value >0.05). In a more recent review that assessed the quality of guidelines in a variety of health topics, it was reported that the quality has improved over time, in contrast to our finding [93]. Since it is a review of many health topics, we are uncertain if the quality of guidelines might have improved in these topics, but not in osteoporosis. This lack of improvement in osteoporosis guidelines should be examined, we think that this perhaps because of the lack of studies with direct evidence on screening for osteoporosis which was reported in many guidelines (NICE 2012[9], and USPSTF 2011[58]).

In comparing the AGREE II tool with the IOM standards; the AGREE II was more comprehensive, as it covered implementation and dissemination issues of the guidelines, while IOM did not cover this area. Both instruments identified lack of compliance in domains relate to multidisciplinary development group composition with involvement of patients and public representatives. Both tools showed that the influence of funding body, and conflict of interests, is falling short in most guidelines as very few CPGs met these criteria.

We used certain criteria to identify high quality guidelines; AGREE II identified less high quality guidelines compared to IOM. By examining the items or criteria for domains of rigor of development and systematic review methodology in the four extra high quality guidelines; identified by IOM; we found that the AGREE II gave lower scores for these guidelines. This is because the AGREE II has more detailed quality items for this section, While, the IOM has fewer criteria in this section, which have resulted in higher scores for the guidelines. This may have important implications for clinicians and stakeholders, in deciding which guideline to implement based on using one of the two tools to assess the quality and rigor of the guidelines.

Our systematic review emphasizes the variability in the use of the different grading systems to aggregate the level of evidence and to rate the strength of recommendations. Establishing the level of evidence that underlies the recommendations is essential in guideline development. Without clarity of the system of evidence that is used, guidelines users cannot decide whether recommendations are built on strong evidence or weak evidence. Additionally, determining the strength of recommendations influences the applicability and implementation of the guideline. Different frameworks or grading systems were developed, yet, the Grading of Recommendations, Assessment, Development and Evaluation (GRADE) system is considered one of the best now, and has been adopted by many organizations [20]. Surprisingly nine guidelines did not use any system for level of evidence or strength of recommendation, and only seven guidelines used the GRADE system (Table 6).

The content analysis of selected areas of recommendations for screening of individuals without previous fractures revealed a considerable variability. This variability did not differ between high or low quality guidelines but mainly differed by region or country. In terms of their approach in screening and using BMD testing or risk assessment first, there was a huge variability between guidelines in this area. The other conflicting area relates to choosing the intervention threshold for those at risk of fracture. Some guidelines use bone mineral density T-score diagnostic criteria. Others use BMD testing with FRAX scores and not solely the T-score results, and this approach is the most adopted in more recent guidelines.

Concordant recommendations were found for BMD testing sites.

We expect some variation between guideline recommendations since it is based on country specific data and cost-effectiveness. However, we found even in the same country guideline recommendations differ. For instance; in Canada; British Columbia CPGs recommend using FRAX before DXA [44], while in Ontario DXA is recommended before FRAX [40], and in Alberta, the use osteoporosis self assessment tool to decide the need for DXA [70]. The lack of uniformity between guidelines, probably creates confusion for the clinicians, and may subsequently affects adherence to the guidelines and diminish quality of care to patients.

### Study limitations and strengths

There are many limitations to this review. First; we included only CPG in English, so guidelines in other languages were not assessed. Second, the AGREE website suggests 2–4 number of reviewers, and four is preferable to increase the reliability of the study. Third; in scoring the AGREE II, only the published information about the guidelines were used in assessment, i.e. we didn’t look at the methodology documents of the organizations, which could have been on their websites. Thus, we may have underscored some domains. Fourth, assessing quality using the IOM standards was challenging because we could not find any suitable published methodology that was used in previous studies, and very few studies have used these standards to assess quality of guidelines (discussed in methods section). Thus, we are not certain about the validity of our methodology. This also may affect the results of comparison between the AGREE II and the IOM standards, which as a result of our IOM methodology, could be liable for bias.

The main strength of this review is that we assessed the quality of osteoporosis screening guidelines over 14-year period to determine changes in guideline quality over time. Our search was systematic and comprehensive including all the general databases, guideline websites and major guideline developer groups, and by hand searching the references of all identified guidelines. Another strength, is that we used two recognised tools to assess guideline quality and compared the results from both tools which to our knowledge was done only by one study [36]. We had a good interrater agreement between the two reviewers which increase the reliability of the study. The AGREE II tool does not assess the content of the recommendations of the guidelines, therefore, another strength is that we examined the content of the recommendations and provided a summary of comparison between the guidelines. In addition, we summarised the different grading systems for level of evidence and strength of recommendations.

## Conclusion

The AGREII and IOM defined quality of CPGs for screening of osteoporosis is variable, and there is a considerable room to improve the guideline development process in this field as well as the reporting of guideline development. Guideline developers should develop their guidelines paying attention to the criteria and standards included in the AGREE II instrument and the IOM standards for trustworthy guideline. The reporting of applicability considerations of the guideline and editorial independence areas appear week. The inclusion of patients, economists, and, knowledge translation experts as well as other stakeholders should be considered as a mean of improving the quality of guidelines and their likelihood of implementation. The lack of consensus on specific guideline recommendations for osteoporosis screening is problematic and creates confusion for clinicians and patients about what exactly is best practice.

## Supporting information

S1 Table Key words used for systematic search. (DOCX)

S2 Table AGREE II tool. (DOCX)

S3 Table IOM standards for Trustworthy Clinical Practice Guidelines. (DOCX)

S4 Table Mapping IOM standards to the AGREE II domains. * Items in bold font don’t have a match in the other tool.
(DOCX)

S5 Table Guidelines with Institute of Medicine Standards (Yes ‘Y’, No ‘N’). (DOCX)
