# Correction: Screening for osteoporosis: A systematic assessment of the quality and content of clinical practice guidelines, using the AGREE II instrument and the IOM Standards for Trustworthy Guidelines

**DOI:** 10.1371/journal.pone.0216196

**Published:** 2019-04-24

**Authors:** Lamia M. Hayawi, Ian D. Graham, Peter Tugwell, Said Yousef

The fourth author’s name appears incorrectly. The correct name is Said Yousef. The correct citation is: Hayawi LM, Graham ID, Tugwell P, Yousef S (2018) Screening for osteoporosis: A systematic assessment of the quality and content of clinical practice guidelines, using the AGREE II instrument and the IOM Standards for Trustworthy Guidelines. PLoS ONE 13(12): e0208251. https://doi.org/10.1371/journal.pone.0208251

The Funding statement is incorrect. The correct Funding statement is as follows: The publication of this study was supported by the Canadian Institutes of Health Research (CIHR): CIHR Foundation grant (FDN #143237), and the University of Ottawa library.

## References

[1] Hayawi LM, Graham ID, Tugwell P, Yousef Abdelrazeq S (2018) Screening for osteoporosis: A systematic assessment of the quality and content of clinical practice guidelines, using the AGREE II instrument and the IOM Standards for Trustworthy Guidelines. PLoS ONE 13(12): e0208251 10.1371/journal.pone.0208251 30521556 PMC6283636

