# Supplementary material for: Screening for osteoporosis: A systematic assessment of the quality and content of clinical practice guidelines, using the AGREE II instrument and the IOM Standards for Trustworthy Guidelines
Source: PLoS One. 2018 Dec 6;13(12):e0208251. doi: 10.1371/journal.pone.0208251 (PMC6283636; doi:10.1371/journal.pone.0208251)
Supplement: S1 Table — (DOCX) [file pone.0208251.s001.docx]

**S1 Table: Key words used for systematic search**

Database: Ovid MEDLINE(R) In-Process & Other Non-Indexed Citations and Ovid MEDLINE(R) <1946 to Present>

Search Strategy:

--------------------------------------------------------------------------------

1 exp clinical pathway/ (5775)

2 exp clinical protocol/ (154284)

3 exp consensus/ (8029)

4 exp consensus development conference/ (11486)

5 exp consensus development conferences as topic/ (2739)

6 critical pathways/ (5775)

7 exp guideline/ (30701)

8 guidelines as topic/ (37891)

9 exp practice guideline/ (23568)

10 practice guidelines as topic/ (102001)

11 health planning guidelines/ (4378)

12 (guideline or practice guideline or consensus development conference or consensus development conference, NIH).pt. (39834)

13 (position statement* or policy statement* or practice parameter* or best practice*).ti,ab,kf,kw. (22878)

14 (standards or guideline or guidelines).ti,kf,kw. (89239)

15 ((practice or treatment* or clinical) adj guideline*).ab. (30162)

16 (CPG or CPGs).ti. (5681)

17 consensus*.ti,kf,kw. (20021)

18 consensus*.ab. /freq=2 (20234)

19 ((critical or clinical or practice) adj2 (path or paths or pathway or pathways or protocol*)).ti,ab,kf,kw. (16533)

20 recommendat*.ti,kf,kw. (33459)

21 (care adj2 (standard or path or paths or pathway or pathways or map or maps or plan or plans)).ti,ab,kf,kw. (42022)

22 (algorithm* adj2 (screening or examination or test or tested or testing or assessment* or diagnosis or diagnoses or diagnosed or diagnosing)).ti,ab,kf,kw. (5860)

23 (algorithm* adj2 (pharmacotherap* or chemotherap* or chemotreatment* or therap* or treatment* or intervention*)).ti,ab,kf,kw. (7141)

24 treatment guidelines.mp. [mp=title, abstract, original title, name of substance word, subject heading word, keyword heading word, protocol supplementary concept word, rare disease supplementary concept word, unique identifier] (8345)

25 or/1-24 (519830)

26 exp Osteoporosis/ (52289)

27 osteoporosis.mp. (76133)

28 exp Bone Density/ (49627)

29 BMD.mp. [mp=title, abstract, original title, name of substance word, subject heading word, keyword heading word, protocol supplementary concept word, rare disease supplementary concept word, unique identifier] (25453)

30 (bone? adj3 (loss* or lose* or losing)).ti,ab. (29496)

31 bone mineral density.ti,ab,kw. (35166)

32 (bone adj2 density).ti,ab,kw. (45159)

33 or/26-32 (127404)

34 25 and 33 (3512)

35 limit 34 to yr="2002 -Current" (2940)

***************************
