# Supplementary material for: Screening for osteoporosis: A systematic assessment of the quality and content of clinical practice guidelines, using the AGREE II instrument and the IOM Standards for Trustworthy Guidelines
Source: PLoS One. 2018 Dec 6;13(12):e0208251. doi: 10.1371/journal.pone.0208251 (PMC6283636; doi:10.1371/journal.pone.0208251)
Supplement: S3 Table — (DOCX) [file pone.0208251.s003.docx]

##### S3 Table: IOM standards for Trustworthy Clinical Practice Guidelines

| STANDARD 1  Establishing Transparency   - 1. The processes by which a CPG is developed and funded should be detailed explicitly and publicly accessible.   STANDARD 2  Management of conflict of interest (COI)  2.1 Prior to selection of the Guideline Development Group (GDG), individuals being considered for membership should declare all interests and activities potentially resulting in COI with development group activity, by written disclosure to those convening the GDG.  • Disclosure should reflect all current and planned commercial (including services from which a clinician derives a substantial proportion of income), non-commercial, intellectual, institutional, and patient/public activities pertinent to the potential scope of the CPG.  2.2 Disclosure of COIs within GDG  • All COI of each GDG member should be reported and discussed by the prospective development group prior to the onset of their work.  • Each panel member should explain how their COI could influence the CPG development process or specific recommendations.  2.3 Divestment  • Members of the GDG should divest themselves of financial investments they or their family members have in, and not participate in marketing activities or advisory boards of, entities whose interests could be affected by CPG recommendations.  2.4 Exclusions  • Whenever possible GDG members should not have COI.  • In some circumstances, a GDG may not be able to perform its work without members who have COIs, such as relevant clinical specialists who receive a substantial portion of their incomes from services pertinent to the CPG.  • Members with COIs should represent not more than a minority of the GDG.  • The chair or co-chairs should not be a person(s) with COI.  • Funders should have no role in CPG development.  STANDARD 3  Guideline development group composition  3.1 The GDG should be multidisciplinary and balanced, comprising a variety of methodological experts and clinicians, and populations expected to be affected by the CPG.  3.2 Patient and public involvement should be facilitated by including (at least at the time of clinical question formulation and draft CPG review) a current or former patient and a patient advocate or patient/ consumer organization representative in the GDG.  3.3 Strategies to increase effective participation of patient and consumer representatives, including training in appraisal of evidence, should be adopted by GDGs.  STANDARD 4  Clinical practice guideline–systematic review intersection  4.1 CPG developers should use systematic reviews that meet standards set by the Institute of Medicine’s Committee on Standards for Systematic Reviews of Comparative Effectiveness Research.  4.2 When systematic reviews are conducted specifically to inform particular guidelines, the GDG and systematic review team should interact regarding the scope, approach, and output of both processes.  STANDARD 5  Establishing evidence foundations for and rating strength of recommendations  5.1 For each recommendation, the following should be provided:  • An explanation of the reasoning underlying the recommendation, including:  • A clear description of potential benefits and harms.  • A summary of relevant available evidence (and evidentiary gaps), description of the quality (including applicability), quantity (including completeness), and consistency of the aggregate available evidence.  • An explanation of the part played by values, opinion, theory, and clinical experience in deriving the recommendation  • A rating of the level of confidence in (certainty regarding) the evidence underpinning the recommendation.  • A rating of the strength of the recommendation in light of the preceding bullets.  • A description and explanation of any differences of opinion regarding the recommendation.  STANDARD 6  Articulation of recommendations  6.1 Recommendations should be articulated in a standardized form detailing precisely what the recommended action is and under what circumstances it should be performed  6.2 Strong recommendations should be worded so that compliance with the recommendation(s) can be evaluated.  STANDARD 7  External review  7.1 External reviewers should comprise a full spectrum of relevant stakeholders, including scientific and clinical experts, organizations (e.g., health care, specialty societies), agencies (e.g., federal government), patients, and representatives of the public.  7.2 The authorship of external reviews submitted by individuals and/or organizations should be kept confidential unless that protection has been waived by the reviewer(s).  7.3 The GDG should consider all external reviewer comments and keep a written record of the rationale for modifying or not modifying a CPG in response to reviewers’ comments.  7.4 A draft of the CPG at the external review stage or immediately following it (i.e., prior to the final draft) should be made available to the general public for comment. Reasonable notice of impending publication should be provided to interested public stakeholders.  STANDARD 8  Updating  8.1 The CPG publication date, date of pertinent systematic evidence review, and proposed date for future CPG review should be documented in the CPG.  8.2 Literature should be monitored regularly following CPG publication to identify the emergence of new, potentially relevant evidence and to evaluate the continued validity of the CPG.  8.3 CPGs should be updated when new evidence suggests the need for modification of clinically important recommendations. For example, a CPG should be updated if new evidence shows that a recommended intervention causes previously unknown substantial harm, that a new intervention is significantly superior to a previously recommended intervention from an efficacy or harms perspective, or that a recommendation can be applied to new populations. |
| --- |
