# Supplementary material for: Screening for osteoporosis: A systematic assessment of the quality and content of clinical practice guidelines, using the AGREE II instrument and the IOM Standards for Trustworthy Guidelines
Source: PLoS One. 2018 Dec 6;13(12):e0208251. doi: 10.1371/journal.pone.0208251 (PMC6283636; doi:10.1371/journal.pone.0208251)
Supplement: S4 Table — * Items in bold font don’t have a match in the other tool. (DOCX) [file pone.0208251.s004.docx]

##### **S4 Table: Mapping IOM standards to the AGREE II domains.**

| AGREE II Instrument | IOM (Standards matching the AGREE II) | |
| --- | --- | --- |
| **Domain 1: Scope and purpose**   1. The overall objective of the guideline is specifically described 2. The health question(s) covered by the guideline is (are) specifically described 3. The population to whom the guideline is meant to apply is specifically described | **No matching standard** | |
| **Domain 2: Stakeholder Involvement**   1. The guideline development group includes individuals from all the relevant professional group 2. The views and preferences of the target population (patients, public, etc.) have been sought. 3. The target users of the guideline are clearly defined. | **Standards 3: Guideline development group composition**  3.1 The GDG should be multidisciplinary and balanced, comprising a variety of methodological experts and clinicians, and populations expected to be affected by the CPG.  3.2 Patient and public involvement should be facilitated by including (at least at the time of clinical question formulation and draft CPG review) a current or former patient and a patient advocate or patient/ consumer organization representative in the GDG.  3.3 Strategies to increase effective participation of patient and consumer representatives, including training in appraisal of evidence, should be adopted by GDGs. | |
| **Domain 3: Rigour of Development**   1. Systematic methods were used to search for evidence. 2. The criteria for selecting the evidence are clearly described. 3. The strengths and limitations of the body of evidence are clearly described. 4. The methods for formulating the recommendations are clearly described. 5. The health benefits, side effects, and risks have been considered in formulating the recommendations. 6. There is an explicit link between the recommendations and the supporting evidence. 7. The guideline has been externally reviewed by experts prior to its publication. 8. A procedure for updating the guideline is provided. | **Standard 4: Systematic review section**  1. CPG developers should use systematic review that meet standers set by the IOM.  2. when SR are conducted specifically to inform the guideline, the team should interact regarding the scope, approach, and output of both processes.  **Standard 5: Establishing Evidence Foundation and rating strength of recommendation**  A clear description of potential benefits and harms.  A summary of available evidence, description of the quality, quantity and consistency of the available evidence. Rating of the level of confidence, and rating of the strength of recommendation, a description and explanation of any differences of opinion regarding the recommendation  **Standard 7: External review**  **1**. External review should comprise a full spectrum of relevant stakeholder, including scientific and clinical experts, organizations, agencies, patents and representative of the public.  2. The authorship of the external reviews submitted by individuals or organizations should be kept confidential unless that protection has been waived by the reviewers.  3. The CDG should consider all external reviewer comments and keep a written record of the rationale of modifying or not modifying a CPGS in response to reviewers’ comments  4. A draft of the CPG at the external review stage or immediately following it should be made available to the public for comment  **Standard 8: Updating**   1. The CPG publication date, date of SR and proposed date for future CPG review should be documented 2. Literature should be monitored regularly following CPG publication 3. CPGs should be updated when new evidence suggests the need for modification of clinically important recommendations. | |
| **Domain 4: Clarity of Presentation**   1. The recommendations are specific and unambiguous. 2. **The different options for management of the condition or health issue are clearly presented**. 3. Key recommendations are easily identifiable | **Standard 6: Articulation of Recommendation**   1. Recommendation should be articulated in a standardized from detailing precisely what the recommended action is and under what circumstances it should be performed 2. Strong recommendations should be worded so that compliance with the recommendations can be evaluated |  |
| **Domain 5: Applicability**   1. **The guideline describes facilitators and barriers to its application.** 2. **The guideline provides advice and/or tools on how the recommendations can be put into practice.** 3. **The potential resource implications of applying the recommendations have been considered.** 4. **The guideline presents monitoring and/or auditing criteria** | **No matching standard** |  |
| **Domain 6: Editorial Independence**   1. The views of the funding body have not influenced the content of the guideline. 2. Competing interests of guideline development group members have been recorded and addressed | **Standard 2: Management of Conflict of interest**  1. Prior to selection of the Guideline Development Group individuals being considered for membership should declare all interests and activities potentially resulting in COI  2. Each panel member should explain how their COI could influence the CPG development process or specific recommendations.  -Members of the GDG should divest themselves of financial investments they or their family members have  2.4 Exclusions: In some circumstances, a GDG may not be able to perform its work without members who have COIs, such as relevant clinical specialists who receive a substantial portion of their incomes from services pertinent to the CPG.  • **Members with COIs should represent not more than a minority of the GDG.**  **• The chair or co-chairs should not be a person(s) with COI.**  • Funders should have no role in CPG development.  **Standard 1: Establishing Transparency**  The process by which a CPGs is developed and funded should be detailed explicitly and publicly accessible |  |

* Items in bold font don’t have a match in the other tool.
