# Supplementary material for: Screening for osteoporosis: A systematic assessment of the quality and content of clinical practice guidelines, using the AGREE II instrument and the IOM Standards for Trustworthy Guidelines
Source: PLoS One. 2018 Dec 6;13(12):e0208251. doi: 10.1371/journal.pone.0208251 (PMC6283636; doi:10.1371/journal.pone.0208251)
Supplement: S5 Table — (DOCX) [file pone.0208251.s005.docx]

**S5 Table: Guidelines with Institute of Medicine Standards (Yes ‘Y’, No ‘N’)**

| IOM Standards | | | | | | | | | | |
| --- | --- | --- | --- | --- | --- | --- | --- | --- | --- | --- |
| **Guideline** | **1.Establishing transparency** | **2.Management of conflict of**  **interest (COI)** | **3.Guideline development group composition** | **4.Clinical practice guideline systematic review intersection** | **5.Establishing evidence foundation for and rating strength of recommendations** | **6.Articulatoin**  **of recommendation** | **7.External review** | **8.**  **Updating** | **Overall**  **Score/8** | **High**  **Quality** |
| **Osteoporosis Canada 2002** ^55^**.** | Y | N | N | Y | Y | Y | N | Y | 5 | ✓ |
| **US Preventive Services Task force 2002** ^56^**.** | Y | Y | N | Y | Y | N | Y | N | 5 | ✓ |
| **Canadian Task Force on Preventive Health Care 2004** ^49^ | N | N | N | Y | Y | Y | N | N | 3 |  |
| **American University of Beirut Medical Center 2005** ^57^**.** | N | N | N | Y | Y | Y | N | Y | 4 |  |
| **Guidelines in Asia 2006** ^48^**.** | N | N | N | N | N | N | N | N | 0 |  |
| **the American College of Physicians guidelines 2008** ^58^**.** | Y | Y | N | Y | Y | Y | N | N | 5 | ✓ |
| **National Osteoporosis Foundation 2008** ^53^**.** | N | Y | N | N | N | N | N | Y | 2 |  |
| **First Update of the Lebanese Guidelines 2008** ^59^**.** | Y | N | N | Y | Y | N | N | Y | 4 |  |
| **Singapore Clinical Guidelines 2009** ^60^ | Y | N | Y | Y | Y | Y | N | Y | 6 | ✓ |
| **Osteoporosis Canada 2010** ^46^**.** | Y | N | Y | Y | Y | Y | N | N | 5 | ✓ |
| **American Association of Clinical Endocrinologist AACE 2010** ^61^ | N | N | N | Y | Y | Y | N | N | 3 |  |
| **The Australien Guidelines 2010** ^44^ | Y | Y | Y | Y | Y | Y | Y | Y | 8 | ✓ |
| **Guideline** | **1.Establishing transparency** | **2.Management of conflict of**  **interest (COI)** | **3.Guideline development group composition** | **4.Clinical practice guideline systematic review intersection** | **5.Establishing evidence foundation for and rating strength of recommendations** | **6.Articulatoin**  **of recommendation** | **7.External review** | **8.**  **Updating** | **Total**  **Score/8** | **High**  **Quality** |
| **South Africa guidelines 2010** ^62^ | Y | N | Y | Y | Y | Y | N | N | 5 | ✓ |
| **Greece National Medicine Agency 2011** ^63^ | N | N | N | N | N | N | N | N | 0 |  |
| **USPSTF 2011** ^64^ | Y | Y | N | Y | Y | Y | Y | N | 6 | ✓ |
| **University of Michigan Health System Guideline 2011** ^65^ | Y | Y | N | Y | N | Y | Y | N | 5 | ✓ |
| **Taiwan osteoporosis practice guidelines 2011** ^66^**.** | Y | Y | N | Y | Y | Y | Y | Y | 7 | ✓ |
| **British Columbia Medical Association 2012** ^50^**.** | Y | Y | Y | N | N | N | N | N | 3 |  |
| **The Malaysian Osteoporosis Society 2012** ^67^**.** | Y | Y | N | Y | Y | Y | Y | Y | 7 | ✓ |
| **NICE guidelines 2012** ^9^ | Y | Y | Y | Y | Y | Y | Y | Y | 8 | ✓ |
| **The Endocrine Society 2012** ^68^ | Y | Y | N | N | Y | Y | Y | N | 5 |  |
| **Institute for Clinical System Improvement guideline, 2013** ^51^**.** | Y | Y | Y | Y | Y | Y | Y | Y | 8 | ✓ |
| **Indian Menopause Society, 2013** ^69^ | N | N | N | Y | N | N | Y | Y | 3 |  |
| **National Foundation of Osteoporosis**  **2014** ^54^ | N | Y | N | N | N | N | N | N | 1 |  |
| **the Society of Obstetricians and Gynaecologists of Canada** ^70^ | N | N | N | N | N | Y | N | N | 1 |  |
| **National Osteoporosis Guideline Group (NOGG)/UK 2014** ^71^ | N | Y | N | N | N | N | N | Y | 2 |  |
| **The Malaysian Osteoporosis Society guideline, 2015** ^72^ | Y | Y | N | Y | Y | Y | N | N | 5 | ✓ |
| **Scottish Intercollegiate Guidelines Network (SIGN) guideline, 2015** ^52^ | Y | Y | Y | Y | Y | Y | Y | Y | 8 | ✓ |
| **2015 Guidelines for Osteoporosis in Saudi Arabia** ^73^**.** | Y | Y | N | Y | N | N | N | N | 3 |  |
| **National Osteoporosis Guideline Group(NOGG)/UK, 2016** ^74^**.** | Y | Y | Y | N | Y | Y | Y | Y | 7 |  |
| **Italian Society for Osteoporosis, Mineral metabolism and Bone Diseases (SIOMMMS) 2016** ^75^ | N | N | N | N | N | N | N | N | 0 |  |
| **Alberta Guidelines 2016** ^76^ | N | N | N | N | N | N | N | N | 0 |  |
| **American Association of Clinical Endocrinologist AACE 2016** ^77^ | N | N | N | Y | Y | Y | Y | N | 4 |  |
